# 2-(2,2-Dibromo­ethen­yl)thio­phene

**DOI:** 10.1107/S1600536811002522

**Published:** 2011-01-22

**Authors:** Sebastien Clément, Laurent Guyard, Michael Knorr, Prisca K. Eckert, Carsten Strohmann

**Affiliations:** a Institut UTINAM UMR CNRS 6213, Université de Franche-Comté, 16 Route de Gray, La Bouloie, 25030 Besançon, France; b Anorganische Chemie, Technische Universität Dortmund, Otto-Hahn-Strasse 6, 44227 Dortmund, Germany

## Abstract

The title compound, C_6_H_4_Br_2_S, represents a versatile building block for the preparation of π-conjugated redox-active thienyl oligomers and metal-mediated cross-coupling reactions. This is due to the presence of an electrochemically active thienyl heterocycle and a reactive dibromo­vinyl substituent, which easily undergoes dehydro­bromination in the presence of *n*-butyl­lithium to afford 2-ethynyl­thio­phene. In the molecule, the alkenyl unit and the thio­phene ring are almost coplanar with an angle of 3.5 (2)° between the normals of the best planes of the thio­phene ring and the vinyl moiety.

## Related literature

The title compound was first prepared in 1980, see: Bestmann *et al.* (1980 ▶). For an alternative synthesis using a Corey–Fuchs reaction, see: Beny *et al.* (1982 ▶). For a structural comparison with 2,2-dibromo­vin­yl[2,2]paracyclo­phane [PCP—C(H)=CBr_2_], (2,2-dibromo­vin­yl)ferrocene [Fc—C(H)=CBr_2_], and 2-thienyl­methyl­enemalononitrile [C_4_H_3_S—C(H)=C(CN)_2_], see: Clément *et al.* (2007*a* ▶,*b* ▶) and Mukherjee *et al.* (1984 ▶), respectively. For recent applications, see: Herz *et al.* (1999 ▶); Rao *et al.* (2010 ▶); Zhang *et al.* (2010 ▶).
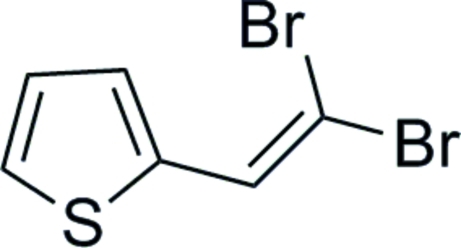


## Experimental

### 

#### Crystal data

C_6_H_4_Br_2_S
*M* *_r_* = 267.97
Monoclinic, 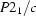

*a* = 9.6843 (19) Å
*b* = 7.2379 (14) Å
*c* = 11.484 (2) Å
β = 109.16 (3)°
*V* = 760.4 (3) Å^3^
*Z* = 4
Mo *K*α radiation
μ = 10.84 mm^−1^
*T* = 173 K
0.4 × 0.4 × 0.2 mm

#### Data collection

Stoe IPDS diffractometer
Absorption correction: numerical (*FACEIT* in *IPDS*; Stoe & Cie, 1999 ▶) *T*_min_ = 0.188, *T*_max_ = 0.658
6492 measured reflections
1667 independent reflections
1444 reflections with *I* > 2σ(*I*)
*R*_int_ = 0.064

#### Refinement

*R*[*F* ^2^ > 2σ(*F* ^2^)] = 0.058
*wR*(*F* ^2^) = 0.139
*S* = 1.04
1667 reflections
82 parameters
H-atom parameters not refined
Δρ_max_ = 1.36 e Å^−3^
Δρ_min_ = −1.73 e Å^−3^

### 

Data collection: *EXPOSE* in *IPDS* (Stoe & Cie, 1999 ▶); cell refinement: *CELL* in *IPDS*; data reduction: *INTEGRATE* in *IPDS*; program(s) used to solve structure: *SHELXS97* (Sheldrick, 2008 ▶); program(s) used to refine structure: *SHELXL97* (Sheldrick, 2008 ▶); molecular graphics: *ORTEP-3* (Farrugia, 1997 ▶); software used to prepare material for publication: *WinGX* (Farrugia, 1999 ▶).

## Supplementary Material

Crystal structure: contains datablocks I, global. DOI: 10.1107/S1600536811002522/im2256sup1.cif

Structure factors: contains datablocks I. DOI: 10.1107/S1600536811002522/im2256Isup2.hkl

Additional supplementary materials:  crystallographic information; 3D view; checkCIF report

## Figures and Tables

**Fig. 1.**
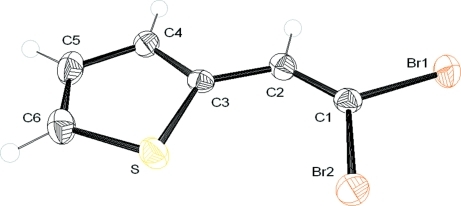
Molecular structure of the title compound with thermal ellipsoids drawn at the 50% probability level.

**Fig. 2.**
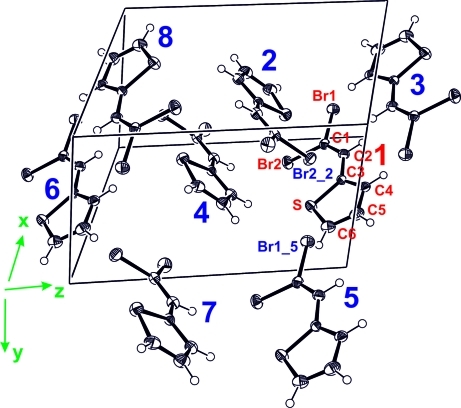
Crystal packing of 2-(2,2-dibromoethenyl)thiophene. Symmetry operations: (1) x, y, z; (2) -x, y - 1/2, -z - 1/2; (3) -x, -y - 1, -z; (4) x, -y + 1/2, z - 1/2; (5) x - 1, y, z; (6) x, y -1, z; (7) x - 1, -y + 1/2, z - 1/2; (8) -x, -y - 1, -z - 1.

## supplementary crystallographic information

### Comment

The title compound (Scheme 1, Fig. 1), which is easily accessible from thiophene-2-carbaldehyde *via* the Corey-Fuchs reaction, has over the last 30 years become a versatile starting material for a variety of organic transformations and a precursor in material science. This interest is due to the conjugation between the electrochemically active thienyl heterocycle with the reactive halogenated olefin moiety. Originally it was used for the preparation of terthiophenes (Beny *et al.*, 1982). Recent applications include Pd-catalyzed cross-coupling reactions (Herz *et al.*, 1999; Rao *et al.*, 2010) as well as the synthesis of imidazo[1,5-α]pyridines (Zhang *et al.*, 2010).

In the course of our interest in developing new π-conjugated dihalovinyl compounds R—C(H)═C*X*_2_ with functional groups (*R* = imine, ferrocenyl, [2,2]paracyclophane) as substrates for oxidative addition reactions across low-valent noble metals, we have recently reported the synthesis and crystal structures of 4-2',2'-dibromovinyl[2,2]paracyclophane (Clément *et al.* 2007*a*) and (2,2-dibromovinyl)ferrocene (Clément *et al.* 2007*b*). With this aim in mind, we also prepared the title compound 2-(2,2-dibromoethenyl)thiophene. A survey of the CSD data base revealed that neither 2-vinylthiophene nor a halogenated derivative of the types [C_4_H_3_S—C(H)═C(H)*X*] or [C_4_H_3_S—C(H)═C*X*_2_] (*X* = halogen) had been structurally characterized yet. The most related molecule found is 2-thienylmethylenemalononitrile [C_4_H_3_S—C(H)═C(CN)_2_] (Mukherjee *et al.*, 1984). In the latter compound, the angle between the normals of the two planar parts of the molecule, the thiophene cycle and the dicyanovinyl moiety, amounts to 3.6 (5)°. In the title compound, the corresponding angle lies in the same range [3.5 (2)°]. A somewhat larger angle of 10.4° has been determined for (2,2-dibromovinyl)ferrocene [Fc—C(H)═CBr_2_] (Clément *et al.*, 2007*b*), whereas in 4-(2',2'-dibromovinyl)[2,2]paracyclophane [PCP—C(H)═CBr_2_] an angle of 51.1° has been observed significantly deviating from coplanarity (Clément *et al.*, 2007*a*). The length of the vinylic C1—C2 double bond [1.335 (7) Å] matches well with those of [PCP—C(H)═CBr_2_] [1.320 (3)°] and [Fc—C(H)═ CBr_2_] [1.318 (4) Å] (Clément *et al.*, 2007*a*; Clément *et al.*, 2007*b*). A similar bond length of 1.353 (5) Å has also been reported for [C_4_H_3_S—C(H)═C(CN)_2_] (Mukherjee *et al.*, 1984).

Bond lenths and angles of the thienyl moiety may be considered as normal and deserve no further comment. The unit cell consists of 4 molecules which are held together by weak interactions only (Fig. 2). These consist of the short Br1—Br2 distance [3.6501 (9) Å, Br1_5-Br2_2] as well as the short distances between Br2 and the carbon atoms of the thiophene ring [3.604 (5) Å, Br2_2-C4; 3.479 (6) Å, Br2_2-C5; 3.624 (5) Å, Br2_2-C6].

### Experimental

Triphenylphosphine (4.20 g, 16.0 mmol), CBr_4_ (5.31 g, 16.0 mmol) and zinc dust (1.05 g, 16.0 mmol) were placed in a Schlenk tube and 40 ml of CH_2_Cl_2_ were slowly added. The mixture was stirred at room temperature for 28 h. Then, 2-thiophenecarboxaldehyde (0.89 g, 8.00 mmol) in CH_2_Cl_2_ (10 ml) was added and stirring was continued for further 2 h. The reaction mixture was extracted with three 50 ml portions of pentane. CH_2_Cl_2_ was added when the reaction mixture became too viscous for further extractions. The extracts were filtered and evaporated under reduced pressure. The crude product was purified by column chromatography on silica gel with CH_2_Cl_2_/petroleum ether (1:4). Slow evaporation afforded white crystals of 2-(2,2-dibromoethenyl)thiophene (yield: 90%). Characterization data have been previously described in the literature. (Beny *et al.*, 1982)

### Refinement

H atoms were refined using a riding model in their ideal geometric positions using the riding model approximation with *U*_iso_(H) = 1.2*U*_eq_(C) for all H atoms.

### Crystal data

**Table:**

C_6_H_4_Br_2_S	*F*(000) = 504
*M**_r_* = 267.97	*D*_x_ = 2.341 Mg m^−^^3^
Monoclinic, *P*2_1_/*c*	Mo *K*α radiation, λ = 0.71073 Å
Hall symbol: -P 2ybc	Cell parameters from 980 reflections
*a* = 9.6843 (19) Å	θ = 2.2–27.0°
*b* = 7.2379 (14) Å	µ = 10.84 mm^−^^1^
*c* = 11.484 (2) Å	*T* = 173 K
β = 109.16 (3)°	Plates, colourless
*V* = 760.4 (3) Å^3^	0.4 × 0.4 × 0.2 mm
*Z* = 4	

### Data collection

**Table:**

Stoe IPDS diffractometer	1444 reflections with *I* > 2σ(*I*)
graphite	*R*_int_ = 0.064
φ scans	θ_max_ = 27.0°, θ_min_ = 2.2°
Absorption correction: numerical (FACEIT in *IPDS*; Stoe & Cie, 1999)	*h* = −11→12
*T*_min_ = 0.188, *T*_max_ = 0.658	*k* = −9→9
6492 measured reflections	*l* = −14→14
1667 independent reflections	

### Refinement

**Table:**

Refinement on *F*^2^	Primary atom site location: structure-invariant direct methods
Least-squares matrix: full	Secondary atom site location: difference Fourier map
*R*[*F*^2^ > 2σ(*F*^2^)] = 0.058	Hydrogen site location: inferred from neighbouring sites
*wR*(*F*^2^) = 0.139	H-atom parameters not refined
*S* = 1.04	*w* = 1/[σ^2^(*F*_o_^2^) + (0.1099*P*)^2^] where *P* = (*F*_o_^2^ + 2*F*_c_^2^)/3
1667 reflections	(Δ/σ)_max_ < 0.001
82 parameters	Δρ_max_ = 1.36 e Å^−^^3^
0 restraints	Δρ_min_ = −1.73 e Å^−^^3^

### Special details

**Table:**

Geometry. All e.s.d.'s (except the e.s.d. in the dihedral angle between two l.s. planes) are estimated using the full covariance matrix. The cell e.s.d.'s are taken into account individually in the estimation of e.s.d.'s in distances, angles and torsion angles; correlations between e.s.d.'s in cell parameters are only used when they are defined by crystal symmetry. An approximate (isotropic) treatment of cell e.s.d.'s is used for estimating e.s.d.'s involving l.s. planes.
Refinement. Refinement of *F*^2^ against ALL reflections. The weighted *R*-factor *wR* and goodness of fit *S* are based on *F*^2^, conventional *R*-factors *R* are based on *F*, with *F* set to zero for negative *F*^2^. The threshold expression of *F*^2^ > σ(*F*^2^) is used only for calculating *R*-factors(gt) *etc*. and is not relevant to the choice of reflections for refinement. *R*-factors based on *F*^2^ are statistically about twice as large as those based on *F*, and *R*- factors based on ALL data will be even larger.

### Fractional atomic coordinates and isotropic or equivalent isotropic displacement parameters (Å^2^)

**Table:**

	*x*	*y*	*z*	*U*_iso_*/*U*_eq_	
Br1	0.90429 (5)	0.69525 (7)	0.15399 (5)	0.0306 (2)	
Br2	0.75506 (5)	0.94572 (7)	0.30330 (4)	0.0290 (2)	
C1	0.7287 (5)	0.7946 (6)	0.1647 (4)	0.0214 (9)	
C2	0.5999 (5)	0.7559 (7)	0.0803 (4)	0.0225 (9)	
H2	0.6061	0.6818	0.0139	0.027*	
C3	0.4530 (5)	0.8068 (6)	0.0720 (4)	0.0188 (8)	
C4	0.3277 (5)	0.7471 (7)	−0.0213 (4)	0.0225 (9)	
H4	0.3305	0.6695	−0.0873	0.027*	
C5	0.1970 (6)	0.8134 (7)	−0.0080 (5)	0.0307 (11)	
H5	0.1026	0.7857	−0.0637	0.037*	
C6	0.2213 (5)	0.9216 (7)	0.0939 (5)	0.0289 (10)	
H6	0.1455	0.9771	0.1174	0.035*	
S	0.40366 (13)	0.94655 (17)	0.17497 (12)	0.0265 (3)	

### Atomic displacement parameters (Å^2^)

**Table:**

	*U*^11^	*U*^22^	*U*^33^	*U*^12^	*U*^13^	*U*^23^
Br1	0.0220 (3)	0.0379 (4)	0.0324 (3)	0.00528 (18)	0.0097 (2)	−0.0004 (2)
Br2	0.0268 (3)	0.0342 (3)	0.0250 (3)	−0.00466 (18)	0.0072 (2)	−0.00800 (17)
C1	0.024 (2)	0.021 (2)	0.0202 (19)	0.0009 (16)	0.0089 (17)	0.0037 (16)
C2	0.024 (2)	0.020 (2)	0.024 (2)	0.0027 (18)	0.0104 (18)	0.0016 (17)
C3	0.024 (2)	0.0157 (19)	0.0183 (19)	0.0008 (15)	0.0095 (17)	0.0015 (15)
C4	0.024 (2)	0.020 (2)	0.024 (2)	0.0029 (17)	0.0083 (18)	0.0041 (17)
C5	0.020 (2)	0.034 (3)	0.036 (3)	−0.0005 (18)	0.005 (2)	0.006 (2)
C6	0.022 (2)	0.028 (2)	0.037 (3)	−0.0003 (18)	0.011 (2)	0.003 (2)
S	0.0256 (6)	0.0287 (6)	0.0277 (6)	0.0006 (4)	0.0121 (5)	−0.0054 (4)

### Geometric parameters (Å, °)

**Table:**

Br1—C1	1.887 (5)	C4—C5	1.408 (7)
Br2—C1	1.878 (5)	C4—H4	0.95
C1—C2	1.335 (7)	C5—C6	1.362 (8)
C2—C3	1.442 (6)	C5—H5	0.95
C2—H2	0.95	C6—S	1.714 (5)
C3—C4	1.397 (7)	C6—H6	0.95
C3—S	1.738 (4)		
			
C2—C1—Br2	124.9 (4)	C3—C4—H4	123.3
C2—C1—Br1	121.3 (4)	C5—C4—H4	123.3
Br2—C1—Br1	113.7 (3)	C6—C5—C4	112.4 (5)
C1—C2—C3	131.4 (4)	C6—C5—H5	123.8
C1—C2—H2	114.3	C4—C5—H5	123.8
C3—C2—H2	114.3	C5—C6—S	112.6 (4)
C4—C3—C2	124.1 (4)	C5—C6—H6	123.7
C4—C3—S	109.8 (3)	S—C6—H6	123.7
C2—C3—S	126.1 (3)	C6—S—C3	91.9 (2)
C3—C4—C5	113.3 (4)		
